# First detection of *Trypanosoma vivax in* small ruminants in the state of Bahia

**DOI:** 10.1590/S1984-29612025053

**Published:** 2025-10-13

**Authors:** Jaqueline Queiroz Amorim Brandão, Hllytchaikra Ferraz Fehlberg, Cássia Matos Ribeiro, Tainara Ferreira Barbosa, Ernesto Souza Oliveira, Rebeca Mabel Oliveira Vieira, Quércia dos Santos Morais, Lucas José Luduverio Pizauro, Wendell Marcelo de Souza Perinotto, Joselito Nunes Costa, George Rego Albuquerque

**Affiliations:** 1 Universidade Estadual de Santa Cruz – UESC, Departamento de Ciências Agrárias e Ambientais, Ilhéus, BA, Brasil; 2 Universidade Federal do Recôncavo da Bahia – UFRB, Centro de Ciências Agrárias, Ambientais e Biológicas, Cruz das Almas, BA, Brasil

**Keywords:** Goats, sheep, semiarid, trypanosomosis, Caprinos, ovinos, semiárido, tripanossomose

## Abstract

The aim of this study was to verify the prevalence of *Trypanosoma vivax* using hematological and molecular techniques and to evaluate possible associated risk factors in goats and sheep. A total of 192 animals from 14 farms in the municipalities of São Domingos and Valente da Bahia were used. Blood was collected from each animal to prepare stained blood smears and verify the presence of *Trypanosoma* spp. trypomastigotes testing and molecular (polymerase chain reaction [PCR], and sequencing). All blood smears were negative for trypomastigote forms of *Trypanosoma* sp. In the molecular analysis, *nested*-PCR detected 57.8% (111/192) of the samples as positive for *Trypanosoma* spp. and 10.9% (21/192) as positive for *T. vivax* using a specific primer. Sequencing indicated 97% – 99% similarity with catL of *T. vivax*. The use of shared needles was significant in the analysis of risk factors (p=0.049). Thus, *T. vivax* is present in small ruminants in Bahia, making it necessary for producers to be careful, especially when sharing needles, to avoid transmission between animals.

## Resumo

O objetivo deste estudo foi verificar a prevalência de *Trypanosoma vivax* usando técnicas hematológicas, moleculares e avaliar possíveis fatores de risco associados em caprinos e ovinos. Foram utilizados um total de 192 animais de 14 propriedades rurais nos municípios de São Domingos e Valente da Bahia. Foi coletado sangue de cada animal para preparação de esfregaços sanguíneos corados e verificação da presença de tripomastigotas de *Trypanosoma* spp. e análise molecular (reação em cadeia da polimerase [PCR], e sequenciamento). Todos os esfregaços sanguíneos foram negativos para as formas de tripomastigotas de *Trypanosoma* sp. Na análise molecular, a PCR detectou 10,9% (21/192) como positivas para *T. vivax* usando um *primer* específico. O sequenciamento indicou similaridade de 97% – 99% com catL de *T. vivax*. O uso de agulhas compartilhadas foi significativo na análise dos fatores de risco (p=0,049). Assim, *T. vivax* está presente em pequenos ruminantes na Bahia, tornando necessário que os produtores tenham cuidado, principalmente ao compartilhar agulhas, para evitar a transmissão entre os animais.

## Introduction

*Trypanosoma vivax* is a protozoan of African origin that causes trypanosomosis, a disease affecting both domestic and wild ruminants. Infections by *T. vivax* have been described in several countries outside of Africa, affecting herds in South and Central America and causing serious economic losses (Benfodil et al., 2020; Bezerra et al., 2023). In Africa, this parasite is transmitted by a biological vector, the tsetse fly of the genus *Glossina* sp. However, in the Americas, as there is no biological vector, studies have suggested that *T. vivax* is transmitted mechanically by hematophagous insects (Raymond, 1990; Otte & Abuabara, 1991) or iatrogenically through the improper use of shared needles, contaminating several animals with infected blood during the application of medications or vaccines (Schmith et al., 2020). Although transplacental transmission is possible, its epidemiological importance remains controversial (Batista et al., 2012; Silva et al., 2013).

*T. vivax* was first diagnosed in Brazil in buffaloes from Pará by Shaw and Lainson in 1972 (Galiza et al., 2011). In the late 1970s and early 1980s, in the northern region, there were several diagnoses of this parasite in cattle, buffaloes and sheep in the states of Pará and Amapá (Serra-Freire, 1981). Through the transport of animals, *T. vivax* was distributed throughout Brazil (Cadioli et al., 2012; Rodrigues et al., 2013; Brito et al., 2017; Bezerra et al., 2018), and the first detection with molecular diagnosis of the parasite in cattle in the state of Bahia was reported in 2021 (Gomes et al., 2021).

The acquisition of newly infected animals and administration of medications using the same needle for several animals are among the main risk factors for the spread of the parasite (Bastos et al., 2020). Infected small ruminants may present with fever, apathy, pale mucous membranes, weakness, progressive weight loss, low milk production, abortion, sudden death, and symptoms common to other parasitic diseases, thus, making the diagnosis and correct treatment difficult (Bonilla et al., 2021).

Diagnosis was based on parasitological, serological, and molecular tests. Parasitological diagnosis exhibits high specificity and low sensitivity but is still widely used in Brazil, with a blood smear stained with Giemsa and subsequent microscopic observation of the parasite (Fidelis et al., 2019; Andrade et al., 2019). However, studies have demonstrated the use of molecular methods such as polymerase chain reaction (PCR) for the diagnosis of *T. vivax*, as these methods indicate the presence of the parasite in the blood of animals (Cadioli et al., 2012) and a better detection rate when compared to parasitological methods, however, their sensitivity is still reduced when there is low parasitemia (Sampaio et al., 2015; Bastos et al., 2020). Although the State of Bahia possesses one of the largest herds of goats and sheep in Brazil, there are still no reports detailing the presence of *Trypanosoma* spp. in these animals. In this study, we aimed to investigate the presence of *T. vivax* in goats and sheep from rural properties in the sisal region of Bahia using molecular methods and hematological tests. We also analyzed potential risk factors for transmission.

## Material and Methods

### Study area and obtaining property information

The present study was approved by the Animal Use Ethics Committee and registered under numbers 2021-47 and 2022-35. The study was conducted in the municipalities of São Domingos (latitude 11°27′56′′ South and longitude 39°31′34′′ West) and in Valente (latitude 11°24′43′′ South and longitude 39°27′43′′ West), sisal region, and semi-arid Bahia, with 192 animals distributed in 14 properties. The samples were collected in the period 2021 and 2022. The properties were selected based on the owner accepting the study and allowing the team to enter the property. The sample size was calculated using the EPI-INFO statistical program version 7.2.5, considering a prevalence of 50%, a sampling error of 7%, and a confidence level of 95% for a population of 25,000 animals. Goats and sheep raised in a semi-intensive system were divided according to age group and sex and then clinically evaluated, associating clinical signs with the presence of the parasite. During sample collection, information related to farm management practices, veterinary care, and knowledge of the disease were obtained through a questionnaire answered by the farm managers. Visual observation of the clinical appearance of the animals in relation to mucous membrane color, hydration, types of feces, and presence of submandibular edema was also performed. The species, age, and sex of each animal was recorded.

### Sample collection and blood smear

Blood (4mL) was collected from each animal by jugular venipuncture using disposable needles (25 × 8 mm) connected to vacuum tubes coated with ethylenediaminetetraacetic acid (EDTA). The samples were stored in a properly identified thermal box, refrigerated, and transported to the Veterinary Parasitology Laboratory of the Federal University of Recôncavo da Bahia (UFRB). Blood smears were prepared, stained using the Giemsa method, and subsequently analyzed under an optical microscope with a 40× objective according to the methodology of Ferreira et al. (1981).

### DNA extraction

Genomic DNA extraction was performed using a 350 µL aliquot of each blood sample using the Easy DNA™ Kit Genomic DNA isolation (Invitrogen™) according to the manufacturer’s recommendations at the Laboratory of Biotechnology and Animal Health of the State University of Santa Cruz (LABSA UESC). Then, the extracted genomic DNA underwent quantification using a NanoDrop 2000 (Thermo Scientific, USA) to verify the quality of the concentration and the purity, and they were subsequently stored and conditioned in a freezer at -20 °C until the molecular analysis was performed.

### Molecular analysis

The DNA samples were subjected to amplification using the Cathepsin L-like gene (TviCatL) specific for *T. vivax*, amplifying approximately 177 bp. The reactions were performed in a PCR mix with a volume of 50 µL that contained 20 to 100ng of genomic DNA, PCR buffer 1×, 1.5 mM MgCl2, 0.2 mM of each dNTP, 100 pmol of each *primer* and 2.5 U of Taq DNA polymerase using the *primers* DTO (5′-TTAAAGCTTCCACGAGTTCTTGATGATCCAGTA-3′) and TviCatL1 (5′-GCCATCGCCAAGTACCTCGCCGA-3′). Positive controls (a *T. vivax* sample kindly provided by Dr. Rosângela Soares Uzeda from the Federal University of Bahia) and ultra-pure water were used as negative controls in the reactions. Amplification conditions in the Proflex PCR system (Applied Biosystems) thermocycler included initial denaturation at 94 °C for 3 min, followed by 35 cycles of denaturation at 94 °C for 1 min, 56 °C for 1 min and extension at 72 °C for 1 min. A final extension at 72 °C for 10 min was then performed (Cortez et al., 2009). The reaction products were subjected to electrophoresis in 1.5% agarose gel, developed with SYBR® safe and photo documented.

### Sequencing

The products obtained from the second PCR were purified using the PureLink PCR Purification kit (Invitrogen) at post-purification concentrations equal to or greater than 30 ng and sent for sequencing to Fundação Oswaldo Cruz-FIOCRUZ-Bahia. The products were subjected to capillary electrophoresis (modified Sanger sequencing) using an ABI 3500XL Genetic Analyzer platform (Applied Biosystems) in both directions. Partial analyses of the chromatograms were performed using FinchTV 1.4.0 software. The Phred-Phrap workflow was then used to obtain the contigs (Machado et al., 2011). The sequences were evaluated using NCBI Basic Local Alignment Search Tool (BLAST) (Altschul et al., 1990). The sequences have been deposited under accession numbers: PQ317119, PQ317120, PQ317121, PQ317122, PQ317123, PQ317124, PQ317125, PQ317126, PQ317127, PQ317128 and PQ317129.

### Statistical analysis

The results of the molecular analyses were associated with variables related to the information collected in the questionnaire. To identify the risk factors associated with infection, bivariate analysis was conducted using the chi-square test or Fisher’s exact test with a significance level of 5% using the EPI INFO program version 7.2.6.0.

## Results

**Figure 1:**
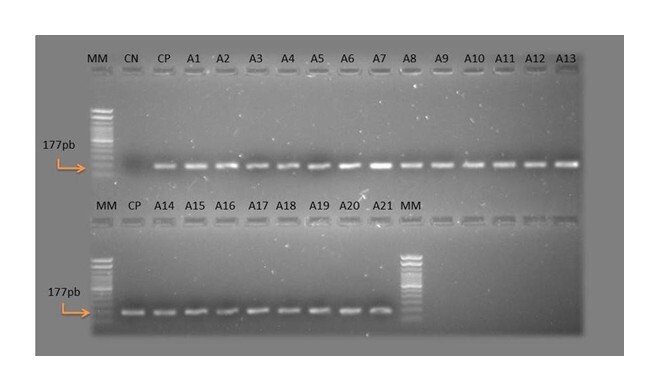
Molecular analysis (PCR and Cathepsin L-like gene) with samples positive for *Trypanosoma vivax*. MM – Molecular marker; CN – Negative control; CP – Positive control; A – Samples positive for *Trypanosoma vivax*; pb – Base pairs.

Of the 192 blood samples collected from small ruminants from 14 farms in the municipalities of São Domingos and Valente, in the Sisaleira region of Bahia, no trypomastigote forms of *T. vivax* were observed in the blood smears. Specific molecular analysis of TviCatL detected 10.9% (21/192) (95% CI: 6.90%–16.23%) positive for *T. vivax* (Figure 1), demonstrating greater sensitivity of molecular analysis in diagnosis. Of the 14 farms studied, six (42.8%) had positive animals (Table 1). In infected animals, clinical signs such as pale mucous membranes, anorexia, dehydration, diarrhea, abortion, reduced body score, submandibular edema and decline in milk production were observed. The rate of infections by *T. vivax* were higher in goats (11.52%, 19/165) than in sheep (7.4%, 2/27); however, the difference was not significant.

**Table 1:** Detection of *Trypanosoma* sp. using PCR of the Cathepsin L-like gene in blood samples from goats and sheep, in the municipalities of Valente and São Domingos, Bahia.

**Property**	**Municipality**	**N^o^ of animals**	**N^o^ of collected animals**	**N^o^ of positive animals (%)**	**Animal species**
					
1	São Domingos	47	19	6 (31.5)	Goat
2	São Domingos	30	18	7 (38.9)	Goat
3	São Domingos	40	19	0 (0.0)	Goat
4	São Domingos	40	19	1 (5.3)	Goat
5	São Domingos	30	10	3 (30.0)	Goat
6	Valente	20	7	2 (28.6)	Sheep
7	Valente	20	9	2 (22.2)	Goat and Sheep
8	Valente	40	10	0	Goat and Sheep
9	Valente	30	10	0	Goat
					Goat and Sheep
10	Valente	25	10	0	Goat
					Goat and Sheep
11	Valente	35	19	0	Goat
12	Valente	30	12	0	Goat
13	Valente	24	10	0	Goat
14	São Domingos	40	20	0	Goat

The contigs obtained in the Phred-Phrap workflow of the *CatL*-like sequences exhibited 97%–99% genetic similarity to the *CatL* of *T. vivax* (accession MT547173.1). In the two samples, it was not possible to obtain contigs; however, analysis of the primer sequences of both revealed 100% similarity to *T. vivax*.

In the analysis of risk factors for *T. vivax* using questionnaire data, only the use of shared needles was significant (p=0.049) (Table 2). All rural properties that were studied were exposed to hematophagous flies (*Stomoxys calcitrans* and/or Tabanidae). Therefore, it was not possible to perform a statistical analysis of this variable. Most owners or those responsible for the properties (11/14, 78.5%) were unaware of the disease. When analyzing the clinical signs that may be related to *T. vivax* infection, an association was observed between positivity and abortion (p=0.02) and decline in milk production (p=0.00002) (Table 3).

**Table 2:** Bivariate analysis of factors associated with *Trypanosoma vivax* infection in goats and sheep, in the municipalities of Valente and São Domingos, Bahia.

Variables	Animals		*χ^2^*	p-value	OR	IC 95%
	Positive (%)	Negative (%)				
Age range						
≤ 01 year	2 (8.0)	23 (92.0)		Ref		
> 01 e < 4 years	12 (13.8)	75 (86.2)		0.907	1.1	0.25 – 7.75
> 4 years	7 (8.7)	73 (91.3)		0.446	1.84	0.46-12.38
Sex						
Male	0 (0.0)	5 (100.0)		1.0*	0	NA-Inf
Female	21 (11.2)	166 (88.8)		Ref		
Shared needle						
Yes	21 (12.9)	141 (8.1)		0.049*	Inf	0.0-NA
No	0 (0.0)	30 (100.0)		Ref		
Have a Fold						
Yes	19 (10.3)	166 (89.7)		0.171*	0.13	0.01 1.64
No	2 (28.6)	5 (71.4)		Ref		
Presence of hematophagous flies**						
Yes	21 (10.9)	171 (89.1)				
No	0	0				
Do Disinfection						
Yes	15 (12.8)	102 (87.2)	0.65	0.41	1.69	0.62 – 4.57
No	6 (8.0)	69 (92.0)	Ref.			
There is a dunghill						
Yes	18 (10.9)	147 (89.1)		0.196*	3,95	0.73-73.44
No	3 (11.1)	24 (88.9)		Ref		
Inclusion of new animals						
Yes	19 (12.6)	132 (87.4)		0.257*	2.91	0.76-19.14
No	2 (4.9)	39 (95.1)		Ref		
Have knowledge about the disease						
Yes	0 (0.0)	42 (100.0)		0.009*	0	NA – Inf
No	21 (14.0)	129 (86.0)		Ref		

* Fisher’s exact test;

** Not performed due to data distribution.

NA – Not available;

Inf – Infinite.

**Table 3:** Analysis of clinical signs associated with *Trypanosoma vivax* infection in goats and sheep, in the municipalities of Valente and São Domingos, Bahia.

Variables	Animals		*χ* ^2^	p-value	OR	IC 95%
	Positive	Negative				
						
Drop in milk production						
Yes	21	98		0.00002*	NA**	4.66-NA
No	0	73				
Abortion			5.14	0.023	3.16	1.24 - 8.07
Yes	13	58				
No	8	113				
Submandibular edema				1.00*	1.17	0.13-10.02
Yes	1	7				
No	20	164				
Pale Mucosa			0.52	0.469	0.56	0.18 - 1.77
Yes	4	50				
No	17	121				

* Fisher's Exact Test;

** NA – Not available.

## Discussion

To our knowledge, this is the first report of *T. vivax* infection in small ruminants in Bahia. The only report of *T. vivax* in this state was in cattle by Gomes et al. (2021); however, reports of producers with trypanosomosis spread throughout Bahia in cattle and small ruminants. No trypomastigote forms of *T. vivax* were observed in the blood smear, and a low detection rate was also reported by Gomes et al. (2021), who obtained only 0.3% positivity in the blood smear. This can be explained by the low sensitivity of parasitological techniques used for identifying infected animals in the chronic phase of infection as described by Fidelis et al. (2019). These animals may have recovered spontaneously from the acute infection and are in the chronic and asymptomatic phases; however, they remain an important source of infection for other ruminants (Bezerra et al., 2023). The blood smear technique exhibits good sensitivity only in infected animals and is evaluated at the beginning of the infection (acute phase), when the animals possess high parasitemia in the blood (Batista et al., 2009).

Molecular PCR analysis detected 10.9% (21/192) of the samples as positive for *T. vivax,* and this is in agreement with previous findings (Fidelis et al., 2019; Hassan-Kadle et al., 2020; Batista et al., 2022). The positivity rate based on the use of *T. vivax*-specific primers in the present study was 10.9%, and a similar result was observed by Ng'ayo et al. (2005) using specific *primers*, where they characterized 5.7% of *T. vivax* infection. As demonstrated in the present study, molecular techniques should be used to minimize the occurrence of false negatives. Batista et al. (2022) reported that this molecular method can detect minimal amounts of *T. vivax* DNA.

In the present study, the positive samples were sequenced, and sequence analysis using BLASTn revealed significant alignments with *TviCatL* sequences, obtaining 97–99% similarity with *T. vivax* present in the National Center for Biotechnology Information (NCBI) database. Cortez et al. (2009) used *TviCatL* in isolates from cattle, goats, and antelopes and demonstrated good specificity in the sequences generated and also confirmed that even with mixed infection of trypanosomatid isolates such as *Trypanosoma congolense*, *Trypanosoma brucei*, and *Trypanosoma theileri*, the gene is a determinant for the species in question. Other studies such as those by Masake et al. (1997) and Ahmed et al. (2013) in which target genes were used for the detection of trypanosomatids, particularly *T. vivax* in ruminants, also showed significant results. These studies reinforce the observation that PCR, as a sensitive technique, provides superior results for detecting *T. vivax* infection in these animals, demonstrating that even in the presence of a small number of trypanosomes, it is possible to detect parasite DNA in the blood compared to detection by other techniques.

The diagnosis of *T. vivax* has already been observed in the study region in cattle through veterinary clinical analysis (Joselito Nunes Costa, personal communication), and as small ruminants and cattle are intercropped in the region, inappropriate management measures can lead to the spread of the parasite between species on the properties. Bastos et al. (2020) reported that the sharing of needles and syringes is an important risk factor for the spread of the parasite a result similar to that in the present study, where the use of shared needles was significant (p=0.049). In all properties with positive samples, the owners claimed to use shared needles, and this is a common practice in the region that may cause the spread of several pathogens among the animals. The presence of hematophagous flies (Diptera: Brachycera) has been cited as a risk factor for the occurrence of the disease (Batista et al, 2008; Dyonisio et al., 2021), among which tabanids are considered transmitters of *T. vivax* in the Americas (Raymond, 1990; Otte & Abuabara, 1991). In all rural properties in this study, the presence of hematophagous flies was noted. Therefore, it was not possible to perform statistical analysis of this variable, but it can be inferred that the presence of these flies increased the chance of transmission of *T. vivax* in the herd.

The affected animals presented clinical signs such as pale mucous membranes, anorexia, dehydration, diarrhea, abortion, body score reduction, submandibular edema and a decline in milk production. Similar signs were observed by Bezerra et al. (2023), who reported intermittent fever, enlarged lymph nodes, reduced body condition scores, pale mucous membranes, apathy, and the birth of small and weak offspring. These signs resemble those of other common diseases in small ruminants such as helminthiasis and eimeriosis, causing owners and professionals to not pay attention to the involvement of goats and sheep in the spread of *T. vivax*. In the present study, the owners reported not being aware of the disease (p = 0.009), and this may lead to confusion regarding clinical signs and ultimately prevent treatment. Pereira et al. (2018) also reported a similar observation.

As a consequence of the infection, an association with abortion (p = 0.02) similar to that observed by Batista et al. (2022) and a decline in milk production (p = 0.00002). Sheep are more frequently infected by *Trypanosoma* spp. than goats under natural conditions, suggesting that goats are more refractory to *Trypanosoma* spp. infections than sheep (Masiga et al., 2002; Ng’ayo et al., 2005). However, in the present study, *T. vivax* infection rates were higher in goats 11.52 (19/165) than in sheep (7.41%, 2/5), corroborating the findings of Hassan-Kadle et al. (2020). The sampling rate was very low in sheep, and this may have influenced the results. Furthermore, the milk production of this region favors the presence of a greater number of goats in the herd than sheep. Small ruminants, particularly goats, are the most important herds in the semiarid region of Brazil. They can be seriously affected by *T. vivax* infection and also be asymptomatic carriers and important sources of *T. vivax* for ruminants in general (Batista et al., 2009).

This study has some limitations that should be considered. Although the sample calculation was carried out based on appropriate parameters, the sampling was not random, i.e. the selection of plots was done for convenience, which affects the extrapolation of results, as the selected plots may not represent the totality of plots in the region. In addition, some variables had a lower number of animals or a complete absence of cases in certain categories, which in some cases led to inaccurate results in the statistical analysis compromising the robustness of the causality analyzes.

## Conclusion

We demonstrated the presence of *T. vivax* in small ruminants in Bahia, and molecular analysis allowed greater sensitivity in the diagnosis of *T. vivax*. The results of the present study suggest that the low adoption of control measures on rural properties in the region, especially in relation to the shared use of needles, may be associated with a risk factor for the spread of the parasite, with infection being a potential cause of economic losses to producers.

## Data Availability

The raw data supporting the conclusions of this article will be made available by the authors upon request. The sequenced amplicons are available on NCBI.

## References

[B001] Ahmed HA, Picozzi K, Welburn SC, MacLeod ET (2013). A comparative evaluation of PCR- based methods for species- specific determination of African animal trypanosomes in Ugandan cattle. Parasit Vectors.

[B002] Altschul SF, Gish W, Miller W, Myers EW, Lipman DJ (1990). Basic local alignment search tool. J Mol Biol.

[B003] Andrade AQ, Mendonça CL, Souto RJC, Sampaio PH, Fidélis OL, André MR (2019). Diagnostic, Clinical and Epidemiological aspects of dairy cows naturally infected by *Trypanosoma vivax* in the states of Pernambuco and Alagoas, Brazil. Rev Bras Med Vet.

[B004] Bastos TSA, Faria AM, Cavalcante ASA, Madrid DMC, Zapa DMB, Nicaretta JE (2020). Comparison of therapeutic efficacy of different drugs against *Trypanosoma vivax* on experimentally infected cattle. Prev Vet Med.

[B005] Batista JS, Bezerra FSB, Lira RA, Carvalho JRG, Rosado AM, Petri AA (2008). Aspectos clínicos, epidemiológicos e patológicos da infecção natural em bovinos por *Trypanosoma vivax* na Paraíba. Pesq Vet Bras.

[B006] Batista JS, Oliveira AF, Rodrigues CMF, Damasceno CAR, Oliveira IRS, Alves HM (2009). Infection by *Trypanosoma vivax* in goats and sheep in the Brazilian semiarid region: from acute disease outbreak to chronic cryptic infection. Vet Parasitol.

[B007] Batista JS, Rodrigues CMF, Olinda RG, Silva TMF, Vale RG, Câmara ACL (2012). Highly debilitating natural *Trypanosoma vivax* infections in Brazilian calves: epidemiology, pathology, and probable transplacental transmission. Parasitol Res.

[B008] Batista JS, Santos WLA, Sousa ACFC, Teófilo TS, Bezerra ACDS, Rodrigues VHV (2022). Abortion and congenital transmission of *Trypanosoma vivax* in goats and ewes in semiarid northeastern Brazil. Res Vet Sci.

[B009] Benfodil K, Büscher P, Abdelli A, Van Reet N, Mohamed-Herif A, Ansel S (2020). Comparison of serological and molecular tests for detection of *Trypanosoma evansi* in domestic animals from Ghardaïa district, South Algeria. Vet Parasitol.

[B010] Bezerra NM, Moura GHF, Araújo HN, Bezerra FSB, Paiva KAR, Costa KMFM (2018). Detection of *Trypanosoma vivax* DNA in semen from experimentally infected goats. Vet Res Commun.

[B011] Bezerra NM, Teófilo TS, Araújo HN, Silva JB, Moura GHF, Costa KMFM (2023). Experimental infection by *Trypanosoma vivax* in goats in the Brazilian semiarid: detection of *T. vivax* DNA in colostrum and assessment of lactogenic transmission. Pesq Vet Bras.

[B012] Bonilla JL, Oliveira JB, Flores B, Jirón W, Sheleby-Elías J (2021). First report of *Trypanosoma vivax* infection in sheep from Nicaragua. Vet Parasitol Reg Stud Rep.

[B013] Brito PD, Lima TS, Oliveira AF, Façanha DAE, Freitas CIA, Braga AP (2017). Evaluation of animal performance, feed intake, and economic losses in sheep experimentally infected with *Trypanosoma vivax.*. Semina: Ciênc Agrár.

[B014] Cadioli FA, Barnabé PA, Machado RZ, Teixeira MCA, André MR, Sampaio PH (2012). First report of *Trypanosoma vivax* outbreak in dairy cattle in São Paulo state, Brazil. Rev Bras Parasitol Vet.

[B015] Cortez AP, Rodrigues AC, Garcia HA, Neves L, Batista JS, Bengaly Z (2009). Cathepsin L-like genes of *Trypanosoma vivax* from Africa and South America – characterization, relationships and diagnostic implications. Mol Cell Probes.

[B016] Dyonisio GHS, Batista HR, Silva RE, Azevedo RCFE, Costa JOJ, Manhães IBO (2021). Molecular diagnosis and prevalence of *Trypanosoma vivax* (Trypanosomatida: Trypanosomatidae) in buffaloes and ectoparasites in the Brazilian Amazon Region. J Med Entomol.

[B017] Ferreira JM, Viana ES, Magalhães LM (1981). Patologia clínica veterinária..

[B018] Fidelis OL, Sampaio PH, Gonçalves LR, André MR, Machado RZ, Wijffels G (2019). Comparison of conventional and molecular techniques for *Trypanosoma vivax* diagnosis in experimentally infected cattle. Rev Bras Parasitol Vet.

[B019] Galiza GJN, Garcia HA, Assis ACO, Oliveira DM, Pimentel LA, Dantas AFM (2011). High mortality and lesions of the central nervous system in trypanosomosis by *Trypanosoma vivax* in Brazilian hair sheep. Vet Parasitol.

[B020] Gomes HCSF, Genipapeiro ILJ, Andrade FT, Barbosa DCV, Pacheco LR, Silva RPB (2021). First detection of *Trypanosoma vivax* in cattle in the state of Bahia, Brazil, based on parasitological and molecular analyses. Semina: Ciênc Agrár.

[B021] Hassan-Kadle AA, Ibrahim AM, Nyingilili HS, Yusuf AA, Vieira RFC (2020). Parasitological and molecular detection of *Trypanosoma* spp. in cattle, goats and sheep in Somalia. Parasitology.

[B022] Machado M, Magalhães WCS, Sene A, Araújo B, Faria-Campos AC, Chanock SJ (2011). Phred-Phrap package to analyses tools: a pipeline to facilitate population genetics re-sequencing studies. Investig Genet.

[B023] Masake RA, Majiwa PA, Moloo SK, Makau JM, Njuguna JT, Maina M (1997). Sensitive and specific detection of *Trypanosoma vivax* using the polymerase chain reaction. Exp Parasitol.

[B024] Masiga DK, Okech G, Irungu P, Ouma J, Wekesa S, Ouma B (2002). Growth and mortality in sheep and goats under high tsetse challenge in Kenya. Trop Anim Health Prod.

[B025] Ng’ayo MO, Njiru ZK, Kenya EU, Muluvi GM, Osir EO, Masiga DK (2005). Detection of trypanosomes in small ruminants and pigs in western Kenya: important reservoirs in the epidemiology of sleeping sickness?. Kinetoplastid Biol Dis.

[B026] Otte MJ, Abuabara JY (1991). Transmission of South American *Trypanosoma vivax* by the neotropical horsefly *Tabanus nebulosus.*. Acta Trop.

[B027] Pereira HD, Simões SVD, Souza FAL, Silveira JAG, Ribeiro MFB, Cadioli FA (2018). Aspectos clínicos, epidemiológicos e diagnóstico da infecção por *Trypanosoma vivax* em rebanho bovino no estado do Maranhão. Pesq Vet Bras.

[B028] Raymond HL (1990). *Tabanus importunus*, vecteur mécanique expérimental de *Trypanosoma vivax* en Guyane Française. Ann Parasitol Hum Comp.

[B029] Rodrigues CMF, Olinda RG, Silva TMF, Vale RG, da Silva AE, Lima GL (2013). Follicular degeneration in the ovaries of goats experimentally infected with *Trypanosoma vivax* from the Brazilian semi-arid region. Vet Parasitol.

[B030] Sampaio PH, Fidelis OL, Marques LC, Machado RZ, Barnabé PA, André MR (2015). Acute-phase protein behavior in dairy cattle herd naturally infected with *Trypanosoma vivax.*. Vet Parasitol.

[B031] Schmith R, Moreira WS, Vassoler JM, Vassoler JC, Souza JEGB, Faria AGA (2020). *Trypanosoma vivax* epizootic infection in cattle from Espírito Santo State, Brazil. Adv Anim Vet Sci.

[B032] Serra-Freire NM (1981). Oiapoque-outro foco de *Trypanosoma vivax* no Brasil. Rev Bras Med Vet.

[B033] Silva TM, Olinda RG, Rodrigues CM, Câmara ACL, Lopes FC, Coelho WAC (2013). Pathogenesis of reproductive failure induced by *Trypanosoma vivax* in experimentally infected pregnant ewes. Vet Res.

